# Spin filtering and switching action in a diamond network with magnetic-nonmagnetic atomic distribution

**DOI:** 10.1038/srep32543

**Published:** 2016-09-07

**Authors:** Biplab Pal, Paramita Dutta

**Affiliations:** 1 Department of Physics, University of Kalyani, Kalyani, West Bengal - 741 235, India; 2 Institute of Physics, Sachivalaya Marg, Bhubaneswar - 751 005, India

## Abstract

We propose a simple model quantum network consisting of diamond-shaped plaquettes with deterministic distribution of magnetic and non-magnetic atoms in presence of a uniform external magnetic flux in each plaquette and predict that such a simple model can be a prospective candidate for spin filter as well as flux driven spintronic switch. The orientations and the amplitudes of the substrate magnetic moments play a crucial role in the energy band engineering of the two spin channels which essentially gives us a control over the spin transmission leading to a spin filtering effect. The externally tunable magnetic flux plays an important role in inducing a switch on-switch off effect for both the spin states indicating the behavior like a spintronic switch. Even a correlated disorder configuration in the on-site potentials and in the magnetic moments may lead to disorder-induced spin filtering phenomenon where one of the spin channel gets entirely blocked leaving the other one transmitting over the entire allowed energy regime. All these features are established by evaluating the density of states and the two terminal transmission probabilities using the transfer-matrix formalism within a tight-binding framework. Experimental realization of our theoretical study may be helpful in designing new spintronic devices.

The ability of manipulating the spin degree of freedom of electrons in transport phenomena has opened up a new field in condensed matter research, called ‘spintronics’^1^ *i.e.*, a spin-based electronics. The aim of this developing field is to design newer and newer electronic devices whose resistances are controlled by the spins of the charge carriers flowing through them^2^. Since its inception in 1996^1^, many spintronic devices acting like, spin-valve^3^, spin-battery^4^, spin-filter^5^, spin- switch^6^,^7^,^8^ etc. have been proposed. However, the actual initiation of the research in this direction was taken a few decades ago. The discovery of first measurements of tunneling magneto-resistance in magnetic tunnel junctions and giant magneto-resistance in Fe/Cr magnetic multilayers boosted intense research on spin-transport phenomena^9^,^10^,^11^,^12^. Devices of this new paradigm of electronics have several advantages like, higher data processing speed, higher integration densities, lower electric power consumption etc. compared to the conventional electronic devices.

Successful incorporation of either the spin degree of freedom to the conventional charge-based electronic devices or the spin alone completely depend on the efficient spin injection, control and manipulation of transport and detection of spin- polarization^1^. Not only semiconductor based devices it is also possible to engineer molecular spintronic devices with various efficiencies and performances depending on the choice of molecule^13^,^14^,^15^,^16^,^17^. However, intensive research is needed to be carried out to make this field more affluent. Therefore, exploring spin-dependent transport phenomena is of great importance today from the perspective of scientific understanding as well as technological applications specially engineering devices with desired magnetic responses. The main reason behind the success of device engineering can be attributed to the recent advances in nano-technology which has also allowed us to design model quantum systems^18^,^19^,^20^ parallely. During the last few decades, such simple looking model quantum systems are drawing attention of the scientific community as they are potential candidates for nano-electronic devices. Along with the spin 1/2 case, the possibility of engineering spin filters for particles with higher spin components using model magnetic quantum device has also been revealed^21^ very recently.

In this present scenario we study spin transmission through a quantum network comprised of an array of diamond-shaped plaquettes connected to each other through the vertices, namely, diamond network, both in absence and presence of magnetic flux threading each diamond plaquette. This one-dimensional lattice (1D) retains the essential features of the T_3_ networks, allowing simple solutions^22^. Transmission phenomena in such diamond networks have already been studied earlier in literature. Various remarkable properties tunable by external parameters have been predicted in this model even in presence of Hubbard interaction^23^,^24^. For instance, in 2009, Sil *et al*. have shown an extrinsic semiconductor-like behavior of such a diamond network depending on suitable choices of the on-site potentials of the atoms at the vertices^25^. Whereas, in 2010 Dey *et al*. have studied the effect of spin-orbit interaction on electron transmission through such diamond network in presence of magnetic flux^26^. But all the atoms of the networks in those works were of non-magnetic types.

However, with the progress of nano-fabrication technique, it is now possible to design various distributions of magnetic and non-magnetic atoms in a single quantum network^27^,^28^,^29^,^30^. Moreover, orientations of the magnetic atoms can easily be tuned by applying external magnetic field resulting angle-dependent transmission spectra. Motivated by this, we explore the two-terminal spin transmission phenomena in a diamond network characterized by magnetic and non-magnetic atomic distribution where, the magnetic ones are situated at the vertices joining each two diamond plaquettes or each boundary plaquette and the lead. Also, each plaquette is penetrated by a uniform magnetic flux perpendicular to the plane of the loop as well as the network. To calculate the transmission probability, transfer-matrix formalism has been used within the framework of tight-binding Hamiltonian. We predict spin-filtering and spin-switching action in this quantum network depending on the magnetic flux, and the strength or the orientation of the magnetic moments.

In what follows, we present our findings. Under the results section, in the first subsection we describe the model and its mapping to one-dimensional chain. In the second subsection we explain the role of the magnetic moments in controlling the energy bands corresponding to the two spin channels leading to the spin filtering effect. Third subsection is devoted to the dispersion profile of the system. The role of the magnetic flux in inducing a switching effect is depicted in the forth subsection. In the fifth subsection we present the phenomena of spin filtering induced by a correlated disorder in the on-site potentials and the magnetic moments. We summarize and conclude about the major findings and utility of our study in the next main section. In the last section we describe the methods used to find the spin-transmission characteristics.

## Results

### The model and mapping to one-dimensional chain

**Figure 1:**
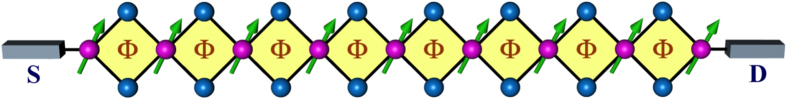
Graphical representation of a diamond array quantum network sandwiched in-between two leads. Schematic diagram of a diamond array network with magnetic-nonmagnetic distribution of atomic sites. The blue spheres represent the non-magnetic sites whereas, the magenta ones correspond to the magnetic sites described by a local magnetic moment (green arrow). The whole network is connected to two non-magnetic semi-infinite ordered leads, namely, source (S) and drain (D).

Let us start by referring to Fig. 1, where we propose a very simple model, an array of diamond-shaped plaquettes attached to two non-magnetic semi-infinite one-dimensional (1D) leads, namely, source and drain. Each plaquette of the array is pierced by an Aharonov-Bohm (AB) flux, Φ (measured in unit of Φ_0_, the elementary flux quantum) perpendicular to the plane of each loop as well as the whole network. The whole network is comprised of magnetic and non-magnetic atoms alternatively placed. More clearly, along the backbone of the diamond array we have considered magnetic sites characterized by a local magnetic moment 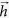, while the top and the bottom lattice sites of each diamond plaquette are occupied by non-magnetic atoms. We write the Hamiltonian of the system using tight-binding framework as,


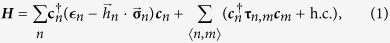


with 〈*n*, *m*〉 denoting the nearest neighbor sites. The creation (annihilation) operator 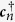 (***c***_*n*_), on-site energy matrix 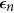 and nearest-neighbor hopping matrix ***τ***_*n*,*m*_ are given by,


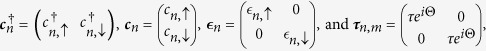


where 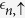 and 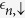 are the on-site energies for the *up* and *down* spin electrons. However, for non-magnetic sites we set 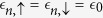. *τ* is the value of the nearest-neighbor hopping integral along each arm of a plaquette. The effect of AB flux^31^ has been incorporated through the phase factor Θ (=2*π*Φ/4Φ_0_). The term 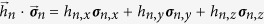 describes the interaction of the spin of the incoming electron with the localized on-site magnetic moment 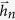 at site *n*, where ***σ***_*n*_ are the set of Pauli spin matrices (***σ***_*x*_, ***σ***_*y*_, ***σ***_*z*_). This term being responsible for the on-site flipping, plays a crucial role in the spin filtering phenomena. The indices ‘↑’ and ‘↓’ correspond to the spin *up* and spin *down* components, respectively. Explicitly, the matrix 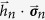 takes the following form^32^,


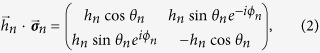


where *θ*_*n*_ and *ϕ*_*n*_ denote the polar and azimuthal angle, respectively.

Now, we can easily map our quasi-one-dimensional chain to an effective one-dimensional chain (similar to the figure in the section Methods) by decimating the top and the bottom non-magnetic sites of each plaquette, and the effect of this mapping is taken care of by the renormalized on-site energy matrices 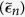 and hopping integrals (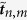) between the magnetic sites. After decimation, the renormalized parameters are of the following form,


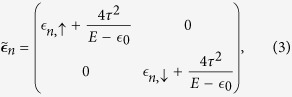



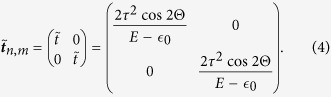


**Figure 2:**
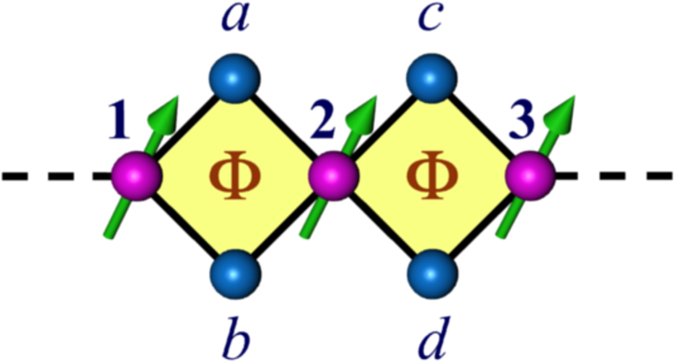
A two-plaquette network. Schematics of a diamond network consisting of two plaquettes, used to show the mapping of the diamond array model to an effective one-dimensional chain.

The decimation scheme to obtain the diagonal matrix elements in Eqs (3) and (4) is as follows, Refer to Fig. 2, we can easily write down the following difference equations for a spin up (↑) electron as,


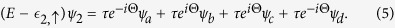



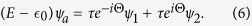



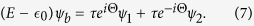



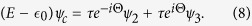



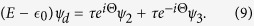


Using Eqs (6)–(9), 19, 20, 21 in Eq. (5) we get,


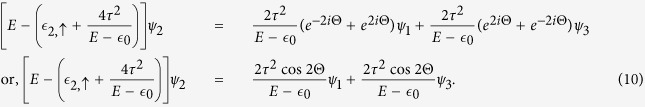


So for an *n*-th site, the form of the renormalized on-site potential for an electron with spin up (↑) state will be 
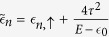
 and the renormalized hopping integral will be 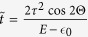. Similarly, we can also have the renormalized parameters for a spin down (↓) electron following the above prescription.

Using the renormalized parameters in Eqs (3) and (4) we write the following set of difference equations corresponding to the *up* and *down* spin channels respectively as,


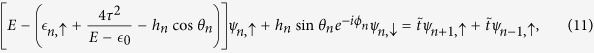



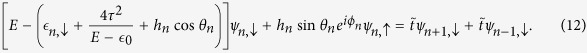


We exploit these two equations to obtain the spectral information corresponding to the two different spin channels. To be mentioned, we set *ϕ*_*n*_ = 0 ∀ *n* throughout our calculation.

Similar to the diamond array, we can also write the tight-binding Hamiltonian for source (drain) by setting 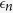 = 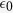, *h*_*n*_ = 0, Θ = 0, *τ* = *t*_*S*(*D*)_ in Eq. (1). To be noted, throughout our calculation, we fix 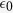 = 0, 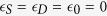, and *t*_*S*_ = *t*_*D*_ = *t*_*SA*_ = *t*_*AD*_ = 3. The source-to-array and array-to-drain couplings are described in terms of *t*_*SA*_ and *t*_*AD*_ respectively.

### Role of substrate magnetic moments in controlling the energy bands – key to spin filter

In this subsection we demonstrate how one can tune the energy bands corresponding to the *up* and *down* spin channels by manoeuvring the amplitudes and the orientations of the magnetic moments, which hold the key to have a spin filtering effect using the diamond network. Let us go back to Eqs (11) and (12). If we set the polar angle, *θ*_*n*_ = 0 ∀ *n*, then we get rid of the cross terms (or hybridizing terms) between the two spin channels as 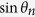 term vanishes irrespective of the azimuthal angle, *ϕ*_*n*_, of the moments. With this choice all the moments will lie along the *z*-direction, being parallel to each other. In addition to this, we also choose 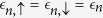. With this choice, Eqs (11) and (12) reduces to the following set of equations,


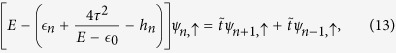



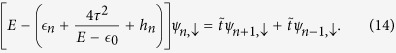


Now, for particular values of 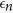, *τ*, 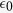, the energy band structures for two different spin channels (spin *up* (↑) and spin *down* (↓)) can easily be tuned just by tuning *h*_*n*_ in absence of magnetic flux (Φ = 0). In order to analyze this fact we have calculated the local density of states (LDOS) corresponding to the spin *up* and spin *down* channels by evaluating the matrix elements of the Green’s function ***G***(*E*) = (*z*^+^***I*** − ***H***)^−1^ in the Wannier basis 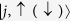 where, *z*^+^ = *E* + *iη* (*η* → 0^+^). The LDOS for the *up* and *down* spin electrons are given by,


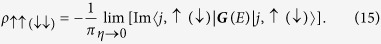


Here we use a real space renormalization group (RSRG) method^33^,^34^,^35^ to obtain the *ρ*_↑↑_ and *ρ*_↓↓_. First we convert the diamond array network into an ‘effective 1D chain’ by decimating the top and bottom non-magnetic sites as stated earlier. Then on-site decimation technique has been used to renormalize the system parameters by folding the chain recursively. As a result of this, the on-site energy matrix and the hopping matrix further get renormalized, and after certain stage of renormalization all the elements of the hopping matrix tends to zero, and finally we are left with a renormalized on-site energy matrix. Using this renormalized on-site energy matrix we obtain the Green’s function, the diagonal elements of which give us the local density of states for the spin *up* and spin *down* channels. The method of renormalization of the on-site energy matrix is shown in the Methods section in details.

**Figure 3:**
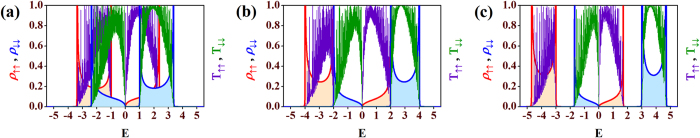
Local density of states (LDOS) and transmission characteristics for spin-up and spin-down electrons. Plots of local density of states (LDOS) and transmission probabilities vs. energy *E* for the spin *up* and spin *down* electrons for three values of *h* with *θ*_*n*_ = 0 (∀*n*) and Φ = 0. The orange shaded plot with red envelope is the LDOS for the spin *up* (↑) states and the light blue shaded plot with blue envelope is the LDOS for the spin *down* (↓) states. The violet lines represent the transmission characteristics for the spin *up* (↑) particles while the green lines correspond to the transmission for the spin *down* (↓) particles for (**a**) *h* = 1, (**b**) *h* = 2, and (**c**) *h* = 3. Also, we set 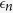 = 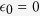 (∀*n*) and *τ* = 1.

**Figure 4:**
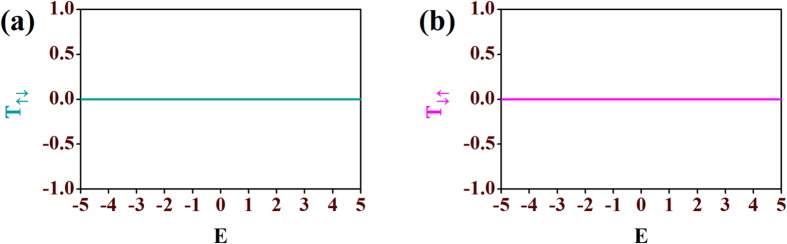
Spin-flip transmission probabilities. Plots of spin-flip transmission probabilities (**a**) *T*_↑↓_ (cyan line) and (**b**) *T*_↓↑_ (magenta line) vs. energy *E*. All other parameters are taken as same as in Fig. 3.

In Fig. 3 we show the effect of the amplitudes of the local magnetic moments *h*_*n*_ on the density of states (LDOS) corresponding to the spin *up* (*ρ*_↑↑_) and spin *down* (*ρ*_↓↓_) channels. We set all the magnetic moments of the network equal to each other (*h*_*n*_ = *h*). By tuning *h* appropriately we can easily tune the energy bands corresponding to the two different electronic spins (↑ and ↓) as evident from the three panels in Fig. 3. We also display the corresponding transmission spectra (*T*_↑↑_ and *T*_↓↓_) for the three cases corresponding to three different values of *h* (=1, 2 and 3) in the same figure. Here, *T*_↑↑_ (*T*_↓↓_) represent the probability of *up* (*down*) electron from the source to transmit as *up* (*down*) electron. To obtain the transmission probabilities of electrons we use the transfer matrix method (TMM) which is described in the last section in detail. Looking at Fig. 3(a) we can say that for *h* = 1, there are overlaps between the spin *up* (↑) and the spin *down* (↓) bands consisting of extended energy levels. There are finite transmission probabilities for each energy level of both the bands. Moreover, due to the overlap between the two spin bands we can have certain energy windows for which both *up* and *down* spin electrons can transmit irrespective of their spin states. Then, we tune *h* to change this energy window common to both the spin states. As soon as we set the value of *h* equal to 2, it is clear from Fig. 3(b) that now there is no overlap between the spin *up* (↑) LDOS and the spin *down* (↓) LDOS. Instead, the bands just touch each other and we get energy regimes for which transmission for only one of the spin states is allowed. For example, if we choose the energy window *E* = 0 to *E* = 2 then, except the boundaries we can have transmission of *up* electrons while it is blocked for the remaining one, *i.e.*, spin *down* electrons. We check it from the transmission-energy characteristics *T*_↑↑_ and *T*_↓↓_. However, at the boundaries the transmissions are not so selective as the bands touch each other at these regions. If we further increase the value of *h*, gaps open up in the energy band as shown in Fig. 3(c) for *h* = 3. So, from the above analysis, it is clear that with an appropriate choice of the substrate magnetic atoms (having the desired value of magnetic moment amplitudes), it is possible to design spin filters with such model quantum systems. To be noted, for all the three cases transmission does not encounter any spin-flip scattering resulting zero *T*_↑↓_ and *T*_↓↑_ as depicted in Fig. 4, where *T*_↑↓_ is the spin-flip transmission probability from a spin *up* (↑) to a spin *down* (↓) state, and *T*_↓↑_ represents the transmission probability for the vice-versa.

Before we end this subsection, we would like to mention that, here we have imposed the condition *θ*_*n*_ = 0 ∀ *n* in order to decouple the two spin channels (Eqs (13) and (14)). Experimental realization of this situation can be easily done by considering a ‘ferromagnetic’ arrangement of the magnetic moments where, all the moments are oriented parallel to each other in the quantum network^36^.

### The dispersion profile

For better understanding of the above discussed phenomena we now study the dispersion profiles for both the spin channels in the diamond network. The mathematical steps are as follow.

We write the wavefunctions *ψ*_*n*,↑_ and *ψ*_*n*,↓_ for *up* and *down* spin-channels in terms of the Bloch waves as,


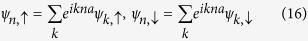


where *a* is the atomic spacing in the effective 1D chain after decimation. In terms of these Bloch waves Eqs (13) and (14) become (for particular value of *k*),


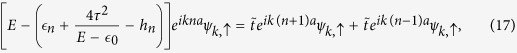



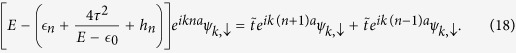


Simplifying these two equations we get two second order polynomials which lead to two solutions for each equation giving the dispersion relations for the two spin channels as,


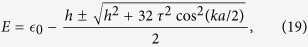


for spin *up* channel, while for spin *down* channel we have,


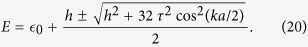


**Figure 5:**
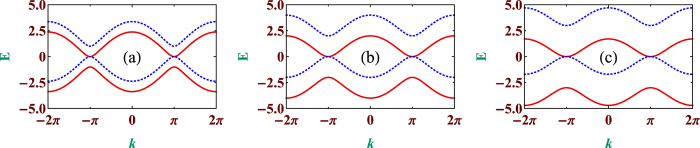
Dispersion curves for the quantum network system. Dispersion profile (*E* − *k*) for the diamond array network. (**a**,**b**,**c**) represent the dispersion profiles for three values of *h*. (**a**) *h* = 1, (**b**) *h* = 2, and (**c**) *h* = 3. Solid red lines and dotted blue lines correspond to spin *up* and spin *down* electrons. Rest of the parameter values are same as in Fig. 3.

To be noted, we set 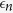 = 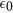, *h*_*n*_ = *h* and *a* = 1. As an illustrative example, we plot *E* vs. *k* in Fig. 5 for some typical parameter values. In Fig. 5, the three panels (a), (b) and (c) correspond to *h* = 1, 2 and 3, respectively. Solid red lines and dashed blue lines represent the *up* and *down* spin bands, respectively. For *h* = 1, it is observed that the spectrum is gapless and two bands corresponding to two different spin channels touch each other at *k* = ±(2*n* + 1)*π*, where *n* = 0, 1, 2, … keeping the periodicity of the profile 2*π* as shown in Fig. 5(a). With the increase of the amplitudes of the magnetic moments from *h* = 1 to *h* = 2, the sub-band separation increases maintaining the central gapless characteristics same as visible in Fig. 5(b). But the degeneracies between the two bands corresponding to a single energy value are not removed completely. For example, let us consider the energy value *E* = 2. Now for this particular choice of the energy value it is easy to understand that there are degeneracies among both the energy bands for *h* = 1. As soon as we increase the amplitudes of the magnetic moments, the band separation increases reducing the degeneracy. After a critical value all the degeneracies among the two spin channels corresponding to *E* = 2 are fully removed. Here, *h* = 2 is the critical value above which we have *up* and *down* spin bands separated from each other except the central region. We have shown this by taking *h* = 3 (see Fig. 5(c)). Instead of degeneracy, now we have a gap around *E* = 2 for *h* = 3. These findings corroborate our earlier study of LDOS and transmission characteristics.

### Magnetic flux induced spin-switch

To reveal the effect of magnetic flux Φ on the energy spectra, we plot LDOS (*ρ*_↑↑_ and *ρ*_↓↓_) and transmission probabilities (*T*_↑↑_ and *T*_↓↓_) as functions of electron energy *E* corresponding to spin *up* and *down* channel, respectively for a particular value of the magnetic flux. Also, we show how one can get a spin-switch using such a simple model. To be mentioned, we do not show *T*_↑↓_ or *T*_↓↑_ further as they are zero for the whole energy window due to the absence of spin-flip scattering.

**Figure 6:**
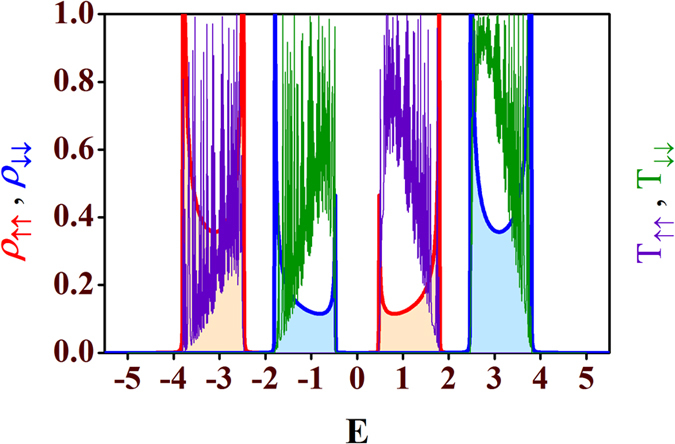
LDOS and transmission characteristics for non-zero flux (Φ = Φ_0_/4). Plot of LDOS and the transmission probabilities vs. the energy *E* for spin *up* (↑) and spin *down* (↓) electrons for a non-zero value of magnetic flux, Φ = Φ_0_**/**4. Here, we choose *h*_*n*_ = *h* = 2. All other parameter values are same as in Fig. 3.

As soon as we turn on the external magnetic flux in each plaquette, the energy bands corresponding to both the spin channels (spin *up* and spin *down*) start to shrink, and gaps (also around *E* = 0) open up in the energy spectrum even for lower values of the moment amplitudes. The central gap can also be observed in the dispersion profiles for a non-zero value of magnetic flux. In Fig. 6 we have shown LDOS and the transmission probabilities as functions of electron energy *E* for Φ = Φ_0_/4 with *h*_*n*_ = *h* being set to 2. We observe that, the energy band widths for the spin *up* (↑) and spin *down* (↓) channels get reduced, and multiple gaps open up in the LDOS spectrum as compared to the case in absence of magnetic flux (Fig. 3(b)).

**Figure 7:**
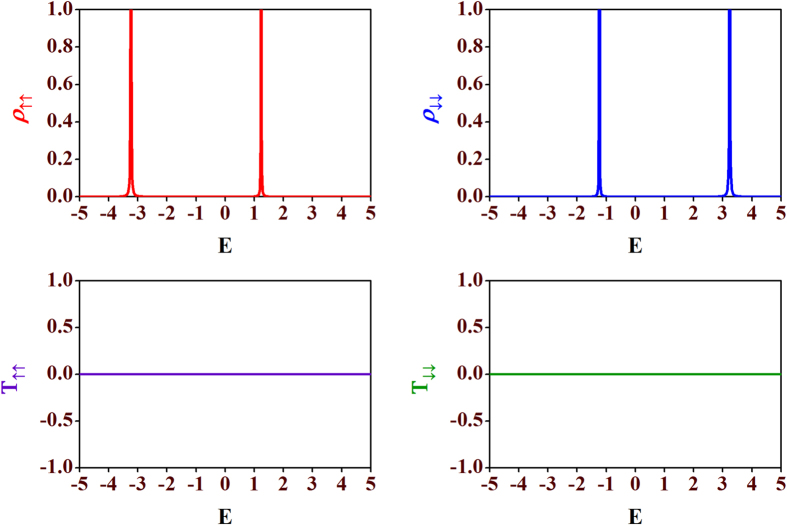
LDOS and transmission probabilities at half flux quantum. Plots of LDOS-energy (upper row) and transmission-energy characteristics (lower row) for spin *up* (↑) (left column) and spin-down (↓) (right column) electrons for half flux quantum, Φ = Φ_0_**/**2. We have set *h*_*n*_ = *h* = 2. All other parameter values are same as in Fig. 3.

**Figure 8:**
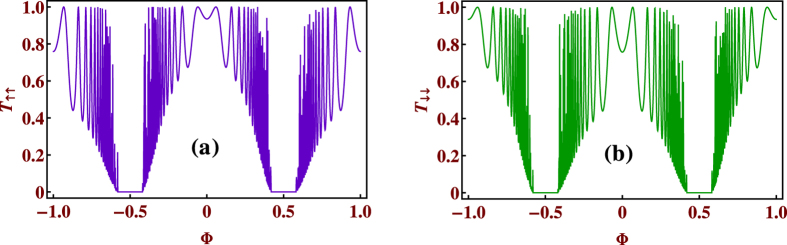
Transmission probabilities vs. magnetic flux at a fixed value of energy. (**a**) Plot of transmission probability *T*_↑↑_ for the spin up (↑) electron with energy *E* = 1, and (**b**) plot of transmission probability *T*_↓↓_ for spin down (↓) electron with energy *E* = −1. All other parameters are taken as same as in Fig. 6.

As we increase the value of the magnetic flux more, the gaps in the energy spectrum become wider, and LDOS for both the spin *up* (↑) and spin *down* (↓) states get narrowed down. Finally, at half flux quantum, *i.e.*, at Φ = Φ_0_/2, we get four extremely localized^37^ pinned states (two for the spin *up* channel and two for the spin *down* channel). They are displayed in Fig. 7. If we look at the corresponding spin transmission spectra for this half flux quantum magnetic flux, we see that there is absolutely no transmission of both the spin states (↑ and ↓) throughout the energy bands. As soon as we tune the value of the magnetic flux from the half flux quantum value (Φ_0_/2), we have finite spin transmission. This behavior of the quantum network makes it possible to predict a magnetic flux induced *switch on-switch off* effect for both the spin states. To make the above discussion more clear, we examine the behavior of the transmission probabilities *T*_↑↑_ and *T*_↓↓_ as a function of the magnetic flux Φ for a particular value of energy. This is shown in Fig. 8. Looking at Fig. 6 we choose two different values of energy corresponding to which we have finite transmission of up or down spin state. Except the region around Φ = Φ_0_/2 we have finite transmission but the magnitude is different for different magnetic flux which is clear from the oscillating nature of the transmission curves. Therefore, just by tuning the external magnetic flux suitably we can trigger a *spin-switch* effect in our diamond network.

### Disorder induced spin filtering

In the earlier subsection we have shown how our model can behave as a spin filter where the filtering effect is induced by the magnetic moment amplitudes *h*_*n*_. Now, in the present subsection we demonstrate how such filtering effect can also be induced by disorder. For this, we choose 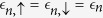.

Looking at Eqs (13) and (14) we see that, if we set 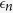 = *h*_*n*_, then the *n*-dependent part of the site energy term for *up* spin channel cancel out while it is present for spin *down* channel. That means putting some on-site disorders in the network lead to disordered chain for the *down* spin channel while it remains an ordered one for the *up* spin channel. Similarly, for 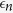 = −*h*_*n*_, exactly opposite phenomenon happens *i.e*., it is disordered chain for the *up* spin channel while ordered for the *down* spin channel with extended states within the energy window 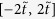. We utilize this fact in order to get the disorder-induced spin filter. Thus we will have transmission for one of the spin channels while the transmission for the other spin channel will be completely blocked.

**Figure 9:**
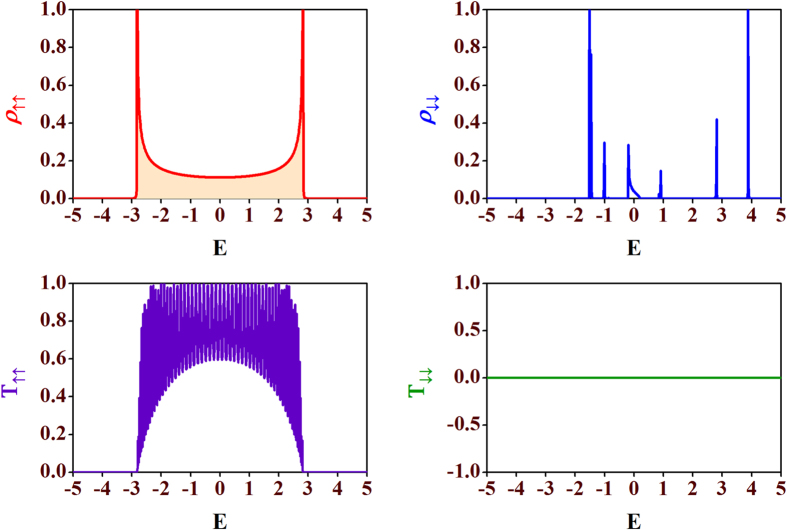
LDOS and transmission probabilities for one set of correlated disorder. Plots of LDOS vs. energy *E* corresponding to spin *up* states, *ρ*_↑↑_ (upper-left) and spin *down* states, *ρ*_↓↓_ (upper-right), and transmission probabilities *T*_↑↑_ (lower-left), *T*_↓↓_ (lower-right) as functions of *E* for the spin *up* and the spin *down* electrons for 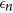 = *h*_*n*_ = *λ* cos(*Qπna*) with *λ* = 3, 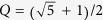, and *a* = 1. We have set Φ = 0, 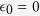 and *τ* = 1.

**Figure 10:**
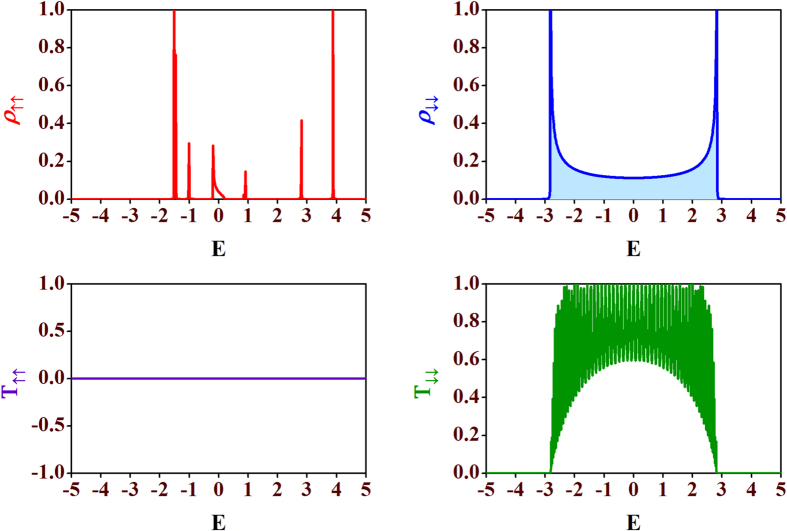
LDOS and transmission probabilities for another set of correlated disorder. Plots of LDOS, *ρ*_↑↑_ (upper-left) and *ρ*_↓↓_ (upper-right), and transmission probabilities *T*_↑↑_ (lower-left), *T*_↓↓_ (lower-right) as functions of electron energy *E* for the spin *up* (↑) and the spin *down* (↓) electrons for 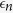 = - *h*_*n*_ = *λ*cos(*Qπna*) with *λ* = 3, 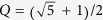, and *a* = 1. We have set Φ = 0, 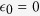 and *τ* = 1.

To verify this we choose the on-site potentials 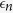 in an Aubry-André variation^38^, *viz.*, 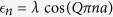 with 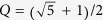, and *a* = 1. Creating these kind of disordered potential in a lattice in a desired way has now become possible especially after the recent progress in experiments in optical lattice using laser beam^39^,^40^,^41^. The results are displayed in Fig. 9. With 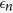 = *h*_*n*_, Eq. (14) correspond to a perfectly ordered chain for the *up* spin channel with all the states being extended in nature (see Fig. 9 (upper-left panel)) and we get a perfect transmission for the spin *up* (↑) states (see Fig. 9 (lower-left panel)). The same correlation between 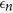 and *h*_*n*_ will make the ‘effective on-site potential’ corresponding to the *down* spin channel equal to 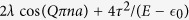 (see Eq. (14)). For a particular choice of *λ* = 3 we show LDOS corresponding to spin *down* (↓) channel. They are critically localized as visible from Fig. 9 (upper-right panel) and we get a zero transmission for the spin *down* (↓) states. The opposite scenario is exhibited in Fig. 10. LDOS corresponding to the *up* and *down* spins are displayed in upper row (left and right, respectively) whereas the transmission spectra are shown in lower row (left and right for *up* and *down* channel, respectively). To be noted, for both the disorder correlations 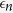 = *h*_*n*_ and 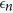 = −*h*_*n*_ spin-flip transmission *T*_↑↓_ and *T*_↓↑_ are zero as there is no spin-flip scattering. So, we can say that by a suitable choice of the on-site potentials and also disorder correlation between the 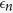 and *h*_*n*_, we can generate a spin filter which allows one of the spin states to pass through the system.

## Discussion

In summary, we have studied spin-dependent transmission through a quantum network with deterministic distribution of magnetic and non-magnetic atomic sites which can act as a magnetic substrate controlled spin filter as well as a magnetic flux induced spintronic switch device. We have shown that by controlling the amplitudes and orientations of the moments of the magnetic sites we can control the energy bands corresponding to the two spin channels which plays the key role in obtaining the spin filtering effect in our model network. By tuning the value of the external magnetic flux suitably we have shown a switch on-switch off effect for the both spin channels which indicates the possibility of designing simple spin switch with such model system. In the last part of the paper we have shown that a correlated disorder in the on-site potentials and in the substrate magnetic moments leads to a different class of spin filter where one can completely block one of the spin channels while making the other one completely transparent.

It is noteworthy to mention that for our model calculation we have taken some particular values of the parameters. With the change of those parameter values except the orientation of the magnetic moments, results will change quantitatively but the qualitative nature of the diamond network will remain same. We have shown all our results for *θ* = 0 *i.e*., all the moments are parallely aligned along *z*-direction. If we deviate the polar angle *θ* from zero to some non-zero value *i.e.*, we rotate the magnetic moments maintaining the ferromagnetic orientation of the magnetic moments then the spin filtering effect will gradually decrease. The amount of decrease will be maximum for anti-ferromagnetic distribution of the magnetic moments. In that situation, spin-filtering effect will no longer exist. Also, to be mentioned, we have set the value of azimuthal angle to zero throughout our manuscript. For any non-zero finite value of the azimuthal angle *ϕ*, all our results will remain unaltered, and both the spin-filtering and spin-switching effects will exist. We have shown our results considering 100 diamond plaquettes in the network. For our proposed model it is needed to take sufficiently large system. The number of diamond plaquettes have to be chosen in such a way that the size effect should not dominate. If we consider a very small system, then the effect of the leads on the transport properties of the system will be large, and the spin-filtering phenomena will be destroyed. But the idea of flux induced spin-switch will work even for a small system as it only depends on the desired value of the magnetic flux for which it occurs.

The mathematical conditions we have imposed to have the interesting results related to spin filtering and spin switching action may be realized by experiments by proper choice of the magnetic materials for the quantum network where the magnetic atoms are subjected to a substrate induced magnetic moments. In the context of magnetic and non-magnetic atomic distribution we can say that the advancement of technology *e.g.*, lithographic technique, chemical deposition and so on, have paved the way of patterning magnetic and non-magnetic atoms in a single network. Various nano-fabrication techniques have made it possible to tailor and control or optimize the magnetic properties also. Creating the whole quantum network with magnetic materials and then engineering it by destroying the magnetic properties of some selective atoms may be another way to design our proposed model.

## Methods

### Renormalization scheme of the on-site energy matrix in the effective one-dimensional chain

**Figure 11:**
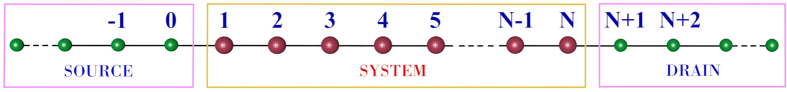
Schematic sketch of the Source-System-Drain bridge. Schematic illustration of the renormalized system coupled in-between two semi-infinite ordered leads.

Refer to the effective 1D system in Fig. 11, we can easily write down the following matrix difference equations,


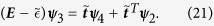



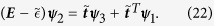



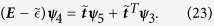


where we have taken 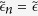 and 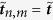, and 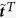 represents the transpose of the matrix 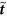. From Eq. (4), it can be easily understood that 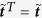. Using Eqs (22) and Eq. (23) in Eq. (21) we get,


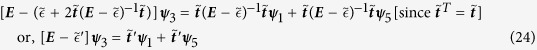


with 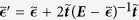 and 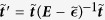 representing the renormalized on-site energy matrix and the renormalized hopping matrix.

### Formulations to obtain the transmission probabilities

In order to get the the transmission probabilities corresponding to the spin *up* and spin *down* channels we have used the transfer matrix method (TMM). We elaborate it here.

Let us start by writing Eqs (11) and (12) in a matrix form as,


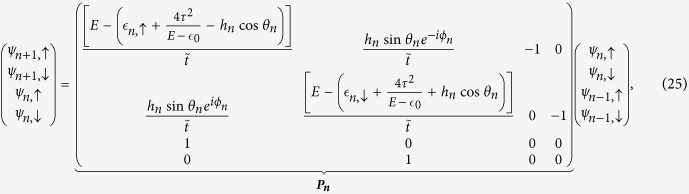


where ***P***_***n***_ is the *transfer matrix* for the *n*-th *renormalized* site.

Now after renormalization we have the diamond array with *N* number of ‘renormalized’ magnetic sites connected in between two semi-infinite non-magnetic leads, *viz.*, source and drain. We display it in Fig. 11. We enumerate the renormalized sites in the array as well as the sites in the two leads as shown in the figure.

So the matrix equation connecting the wave functions of the source-system-drain bridge (see Fig. 11) is given by,


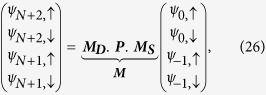


where ***M***_***S***_ and ***M***_***D***_ are the transfer matrices for the source, and drain, respectively. 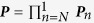, and ***M*** is the total transfer matrix for the source-system-drain bridge.

#### Evaluation of *M*_*S*_

Let us set 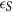 = 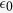 for all the sites in the source. Now, we write the difference equation connecting the wave function amplitudes of the 0-th site with that of the 1-st and (−1)-th sites as,


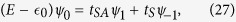


where *t*_*S*_ is the hopping integral value for the sites in the source and *t*_*SA*_ denotes the coupling of the source to the diamond array network. Now in the lead, we can write the incoming wave function for each site as,


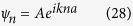


or for particular choice of site it can be taken as,


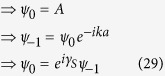


where *γ*_*S*_ = *ka* and 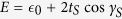. Using Eq. (29) in Eq. (27) it can easily be found that,


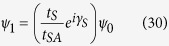


We can reframe Eqs (29) and (30) including the spin indices in the following form,


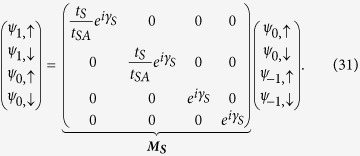


#### Evaluation of *M*_*D*_

Similar to the source we write the difference equation connecting the wave function amplitude of the (*N* + 1)-th site to that of the (*N* + 2)-th and *N*-th sites (taking 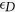 = 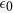) as,


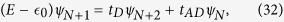


where *t*_*D*_ is the hopping integral in between the sites in the drain and *t*_*AD*_ represents the coupling term.


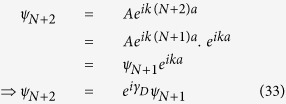


where *γ*_*D*_ = *ka* and 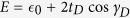. Using Eq. (33) in Eq. (32) it can be easily obtained that,


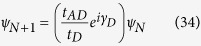


We can easily reframe Eqs (33) and (34) including the spin indices to obtain the following form,


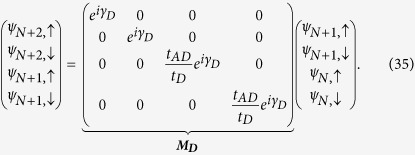


Now, let us consider two cases corresponding to the two spin states *up* and *down* of the incoming electron.

♦ **Scenario 1:** *Incoming electron is spin up*

If the incoming electron is spin *up* then the wavefunction amplitudes in Eq. (26) can be written in terms of the reflection and transmitted amplitudes as,


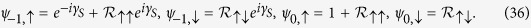



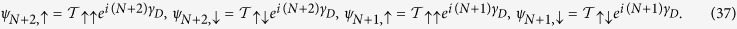


where 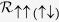 and 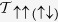 are the amplitudes of the reflected and transmitted electron wavefunctions with spin *up* (↑) projection, which remain in spin *up* (↑) state (or, flip to spin *down* (↓) state) after passing through the system. Putting the above expressions of the wavefunction amplitudes in Eq. (26) we will have the following equation,


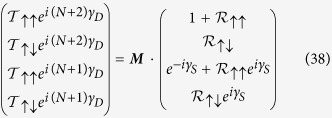


We solve Eq. (38) to have the values of the 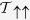 and 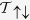. Finally, we obtain the transmission probabilities by using the formulae,


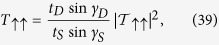



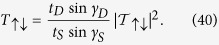


The total transmission probability for spin *up* (↑) particle is given by,


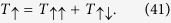


♦ **Scenario 2:** *Incoming electron is spin down*

On the other hand, if the incoming particle from the source has a spin *down* (↓) state then the wavefunction amplitudes in Eq. (26) can be expressed as,


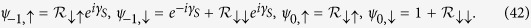



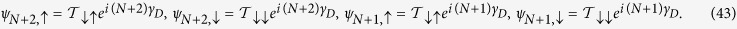


where 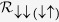 and 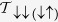 are the amplitudes of the reflected and transmitted electron wavefunctions with spin *down* (↓) projection, which remain in spin *down* (↓) state (or, flip to spin *up* (↑) state) after passing through the system. If we put the above values of the wavefunction amplitudes in Eq. (26) then we will have the following matrix equation,


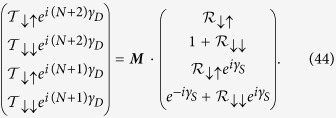


We can easily solve Eq. (44) to get the values of the 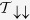 and 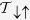. Then finally, we obtain the transmission probabilities by using the formulae,


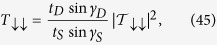



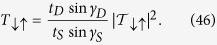


The total transmission probability for spin *down* (↓) particle is given by,


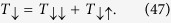


## Additional Information

**How to cite this article**: Pal, B. and Dutta, P. Spin filtering and switching action in a diamond network with magnetic-nonmagnetic atomic distribution. *Sci. Rep*. **6**, 32543; doi: 10.1038/srep32543 (2016).
